# Label-free visualization of lignin deposition in loquats using complementary stimulated and spontaneous Raman microscopy

**DOI:** 10.1038/s41438-019-0153-3

**Published:** 2019-06-01

**Authors:** Nan Zhu, Yifan Yang, Minbiao Ji, Di Wu, Kunsong Chen

**Affiliations:** 1 College of Agriculture & Biotechnology, Zhejiang University, Zijingang Campus, Hangzhou, 310058 People’s Republic of China; 2 State Key Laboratory of Surface Physics and Department of Physics, Human Phenome Institute, Multiscale Research Institute of Complex Systems, Key Laboratory of Micro and Nano Photonic Structures (Ministry of Education), Fudan University, Shanghai, 200433 People’s Republic of China; 3 Zhejiang Provincial Key Laboratory of Horticultural Plant Integrative Biology, Zhejiang University, Zijingang Campus, Hangzhou, 310058 People’s Republic of China; 4 The State Agriculture Ministry Laboratory of Horticultural Plant Growth, Development and Quality Improvement, Zhejiang University, Zijingang Campus, Hangzhou, 310058 People’s Republic of China

**Keywords:** Plant sciences, Metabolomics

## Abstract

The lignification triggered by biotic or abiotic stresses hardens fruits and vegetables and eventually influences their consumer appeal. Extensive prior efforts have been made to unveil the underlying mechanism of flesh lignification, primarily focused on its physicochemical and molecular biological properties. Nevertheless, most of these studies used destroyed and homogenized bulk tissues as analytes; as a result, potentially valuable spatial information was lost. In this study, the deposition of lignin in loquat flesh during lignification was visualized from the tissue level to the single-cell level by combining the advantages of stimulated Raman scattering (SRS) and spontaneous Raman microscopy using label-free in situ molecular imaging. SRS has the advantages of being fast and providing large-area chemical imaging to reveal the spatial heterogeneity of lignin and cell wall polysaccharide distribution in loquat flesh. After 2 days of storage at 0 °C, increased lignins were observed by large-area SRS imaging. In addition, microscopic SRS images of the flesh cells indicated that the increased lignins were trapped in the cell corner (CC) and middle lamella (ML). Furthermore, the compositional and structural features of lignified cells (LCs), CC and ML of loquat flesh were investigated by spontaneous Raman microscopy, and the results showed that the LCs were a combination of lignin, cellulose, and hemicellulose, whereas CC and ML showed only deposited lignin and pectin without cross-linked cellulose and hemicellulose. This result further suggests that the lignins in the CC and ML regions of loquats were later synthesized alone during postharvest storage. This innovative combination of SRS and spontaneous Raman microscopy allows the label-free macroscale and fine chemical imaging of plant cell walls and will enhance our fundamental understanding of the structures and functions of the plant cell wall.

## Introduction

In recent decades, fruits have become an indispensable part of the family diet, and the household consumption of fruit has increased significantly. Textural changes in fruits during postharvest that convey various sensory properties are attracting a great deal of concern and interest from consumers. The most typical textural characteristics of postharvest fruits are softening and lignification (hardening)^1,2^. Fruit softening is a common phenomenon that occurs in many fruits, such as tomatoes^3,4^, kiwifruits^5,6^, apples^7,8^, and peaches^9,10^, during the postharvest phase; the progression of fruit softening is related to the progressive depolymerization and solubilization of cell wall components and the loss of the cell structure. However, fruit lignification leading to increased fruit or fruit pericarp firmness is an unusual phenomenon that occurs in several fruits during postharvest^11,12^. In mangosteen fruits, pericarp lignification is triggered by impact damage, which enhances the incorporation of phenolics into the lignin and increases the pericarp firmness^13^. Another notable case is the substantial lignification of loquat flesh caused by chilling stress during postharvest storage^14,15^. Loquat fruits are harvested during a high-temperature and high-humidity season, and they suffer rapid senescence and deterioration. Low-temperature storage can maintain the fruit quality and extend the shelf life of loquats but results in substantial flesh lignification. Previous studies have shown that the “Luoyangqing” loquat suffers chilling injury when it is stored at 0 °C^16,17^. Loquat fruits suffering from lignification exhibit an increase in lignin content and firmness, along with the presentation of some physiological disorders, including a stuck peel and leathery and juiceless pulp. The lignification of the fruit will inevitably influence the fruit texture, affect the fruit storability and quality, and eventually reduce consumer acceptance^1^.

Lignification involves the progressive biosynthesis and deposition of lignin as mediated by related enzymes in plants^18,19^. During lignification, monolignols (coniferyl alcohol, sinapyl alcohol, and p-coumaryl alcohol) are synthesized in the cytoplasm through the general phenylpropanoid and monolignol-specific pathways and then transported to the lignification zone for radical polymerization^19,20^. The reduction of coniferyl aldehyde through the action of coniferyl aldehyde dehydrogenase is considered the final step in the biosynthesis of coniferyl alcohols (lignin-CAlc) in lignin^19^. The synthesized lignin in plants can provide mechanical strength for vascular and support tissues such as vessel elements, xylem tracheids, and sclereid cells. Researchers have revealed that fruit firmness is closely related to the lignin content^21,22^. For example, a positive correlation between firmness and increased lignin (*r* = 0.95**) was found in “Luoyangqing” loquat fruits^15^.

Researchers have been trying to uncover the mechanism underlying fruit lignification for decades^14,23,24^. In addition to studying the basal physicochemical characteristics, including fruit firmness and lignin content, they have performed extensive studies on the molecular biological characteristics of fruit lignification^11,25,26^. The lignin biosynthesis-related genes (*PAL, 4CL*, and *CAD*) and their transcription factors (*NAC* and *MYB*) have been actively studied during lignification in fruit^14,25,27^. Nevertheless, most of these physicochemical and molecular biological analyses were based on homogenized bulk tissues obtained via destructive sampling, which provided only the average content variations. These results indicate the presence and abundance of target substances but do not provide information on their spatial localization in fruit flesh at the cell level. In fact, cell-level mapping of the spatial localization of target substances has great significance for revealing the phenotypic heterogeneity among the massive cells of fruit flesh. This visible phenotypic heterogeneity, in combination with single cell-omics analysis, can provide novel insights into deciphering the mechanisms underlying fruit lignification. To the best of our knowledge, few studies have been conducted on the direct visualization of lignin accumulation in fruits during lignification.

Over the past two decades, Raman-based imaging techniques have shown great potential in the direct visualization of the distribution, topochemical and structural dynamic changes in lignin in plant tissues via a label-free and in situ approach^28–30^. Of the possible techniques, confocal spontaneous Raman microscopy and stimulated Raman scattering (SRS) microscopy offer the most options. Spontaneous Raman spectroscopy is more fundamental than SRS and reveals the full intrinsic fingerprint spectra, covering the entire window of molecular vibrations. The full spectra carry a wealth of spatially resolved information concerning the biochemical composition in the context of the structurally complex mixtures found in plant tissues. Even subtleties in the spectra can be extracted via spectral preprocessing and multivariate analysis^31^. Based on spontaneous Raman hyperspectral data, the simultaneous visualization of multiple components in one view is achieved without staining or labeling^8,32^. Moreover, the band height ratios in the full spectra imply that there is worthwhile information regarding the compositional and ultrastructural features of various substances in the sample^33–35^. For example, the orientation of the cellulose microfibril angle, which is closely related to the architecture of the plant cell wall, can be predicted by surveying the orientation-sensitive cellulose bands^34,36^. In addition, the relative abundance of the different functional groups in lignin can be evaluated by calculating the ratios of their marker bands^33,37^. Despite its advantages in relation to the intrinsic full and informative hyperspectral data and simultaneous visualization of multiple components, spontaneous Raman microscopy inherently suffers from relatively low imaging speed and sensitivity, which limit its application to fast chemical imaging in living systems or large-area imaging in large samples. Fortunately, these problems can be solved by the advent of SRS microscopy, which has significantly greater imaging efficiency than that of spontaneous Raman microscopy. The detection sensitivity of SRS is significantly improved by implementing high-frequency (megahertz) phase-sensitive detection^38^. Moreover, SRS offers a speed advantage by focusing on either a single Raman band or a defined spectral window of target molecules^39,40^. Compared with coherent anti-Stokes Raman scattering, which is another type of coherent Raman scattering technique, SRS shows clear advantages in its background-free and readily interpretable chemical contrast^41,42^. These advantages make SRS a superior alternative for fast and large-area biological chemical imaging.

It is essential to note that SRS and spontaneous Raman scattering complement one another^40^. Combining SRS imaging with spontaneous Raman microscopy allows the researcher to take advantage of the respective advantages of the two approaches to explore the biochemical composition and molecular structure features of the sample from different perspectives. Here, we combined SRS microscopy with spontaneous Raman microscopy to investigate the lignification of loquat fruit during cold storage from the tissue level to the single-cell level. Considering its substantially higher detection sensitivity and acquisition speed, SRS microscopy was used to plot the chemically specific contrast of the tissues and cells of loquat fruits during lignification, from the broad view to the details. Macroscale chemical imaging in tandem with microscopic analysis makes this technique attractive for presenting overall information about the chemical structure and composition of the sample, and thus the overgeneralization caused by the narrow field of microvision can be avoided. However, spontaneous Raman microscopy can be used to scrutinize the detailed chemical fingerprints of different morphological zones to visualize multiple components simultaneously. In general, we show that SRS and spontaneous Raman microscopy are complementary potential techniques for the thorough investigation of lignin deposition in loquat fruits during postharvest storage.

## Results

### Simultaneous imaging of cell wall polysaccharides and lignin at both macro- and microscale by SRS microscopy

**Fig. 1:**
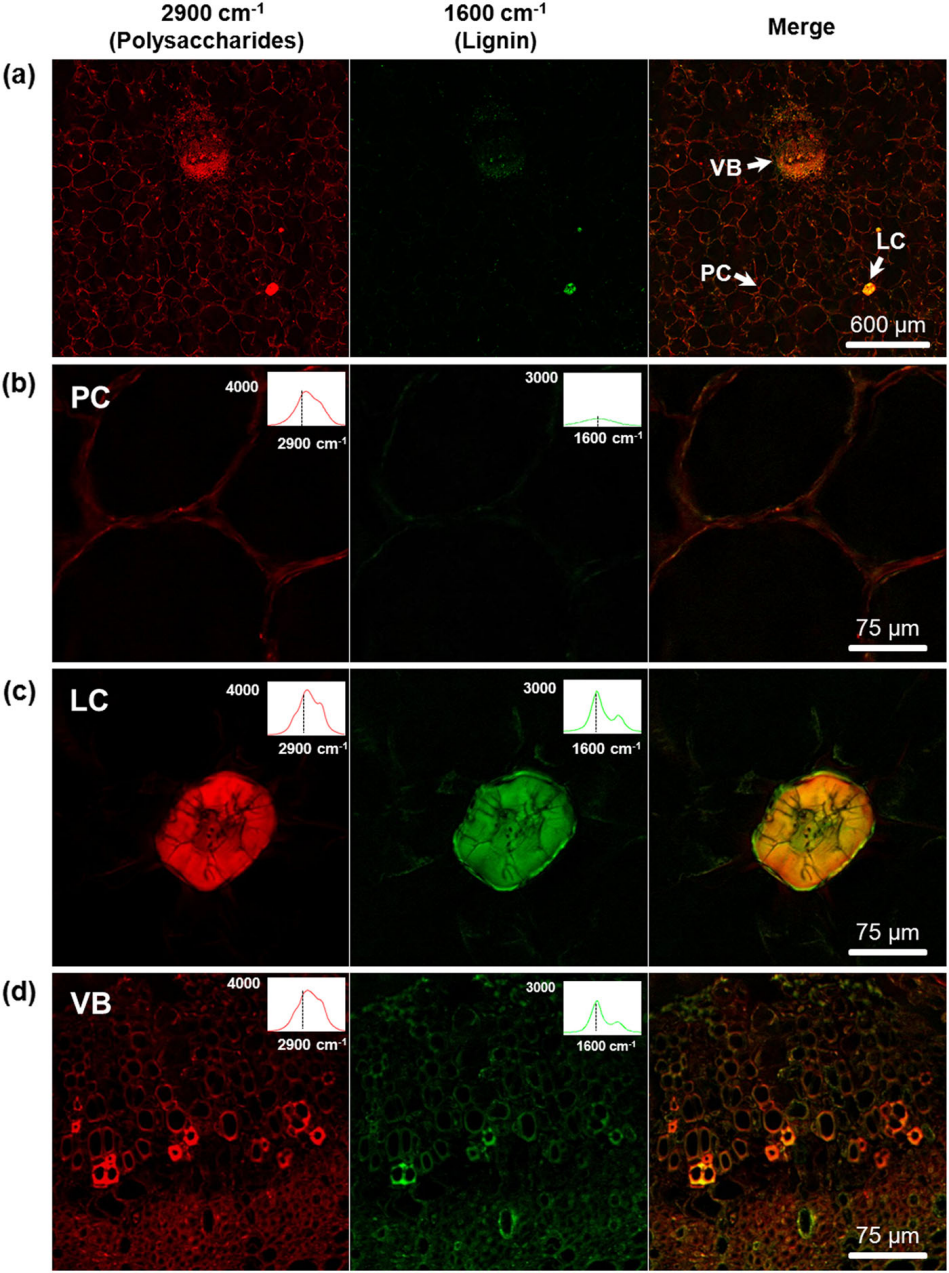
SRS images of cell wall polysaccharides and lignin in loquat flesh at both the macroscale and the microscale. The simultaneous imaging of polysaccharides at 2900 cm^−1^, lignin at 1600 cm^−1^ and their merged images for the macroscale view (**a**), parenchymal cell (**b**), lignified cell (**c**), and vascular bundle (**d**). The upper right corner of each corresponding image shows the extracted spectra from the SRS image stack. PC parenchymal cell, LC lignified cell, VB vascular bundle

Figure 1 shows SRS images of the cell wall polysaccharide and lignin distribution in loquat flesh at both the macroscale (Fig. 1a) and the microscale (Fig. 1b–d). Using SRS imaging, the distributions of cell wall polysaccharides and lignin in loquat flesh can be observed simultaneously with a large field of view (Fig. 1a). Furthermore, in combination with microscopic chemical imaging, three types of cells were distinguished, i.e., the parenchymal cell (PC), lignified cell (LC), and vascular bundle (VB), which were the primary cell types found in loquats. PC, LC, and VB were all detected as SRS signals of cell wall polysaccharides at 2900 cm^−1^, which is generated by C–H stretching. The LC shows higher SRS signals for cell wall polysaccharides than in the PC and VB. The intensity of the SRS signal is linearly dependent on the analyte concentration^38,43^. Therefore, the cell wall polysaccharide content in the LC is higher than that in the PC and VB in loquats. For the VB, some cells show high SRS signals and thus high contents of cell wall polysaccharides. These cells might be the xylem vessels in loquat. The SRS signal at 1600 cm^−1^ corresponding to the aromatic-ring breathing vibration of lignin was used to plot the distribution of lignin in the loquats. The LC and some cells in the VB show higher lignin signals and thus have high lignin contents. In contrast, the PC shows little lignin signal, indicating its limited lignin content.

To confirm the veracity of the SRS signal attribution, the Raman spectra were extracted based on the acquired image stacks. The upper right corner of each SRS chemical image (Fig. 1b–d) shows the corresponding extracted Raman spectrum. As shown in Fig. 1b–d, the extracted spectra from the 2900 cm^−1^ signal of the PC, LC, and VB are all typical of cell wall polysaccharides, showing a shoulder at 2930 cm^−1^. This result confirms that the SRS signal at approximately 2900 cm^−1^ is attributable to cell wall polysaccharides in loquats. For the SRS images generated at 1600 cm^−1^, the extracted spectra based on the image stack indicate differences among the PC, LC, and VB. The extracted spectrum from the 1600 cm^−1^ PC signal shows a low and broad band without any typical characteristics of lignin. Although the 1600 cm^−1^ SRS signal of the PC is detected, this signal is more likely attributable to interference by other factors. The LC and VB signal channels exhibit typical characteristic spectral features for lignin, showing a shoulder at approximately 1660 cm^−1^ (Fig. 1c, d). The above results indicate that SRS imaging is a powerful tool for the label-free chemical imaging of loquat flesh with both large-area and cellular spatial resolution. The label-free chemical contrast among different cell types in loquat flesh can be achieved by SRS imaging.

### SRS imaging of changes in cell wall polysaccharides and lignin in loquat flesh during postharvest storage

**Fig. 2:**
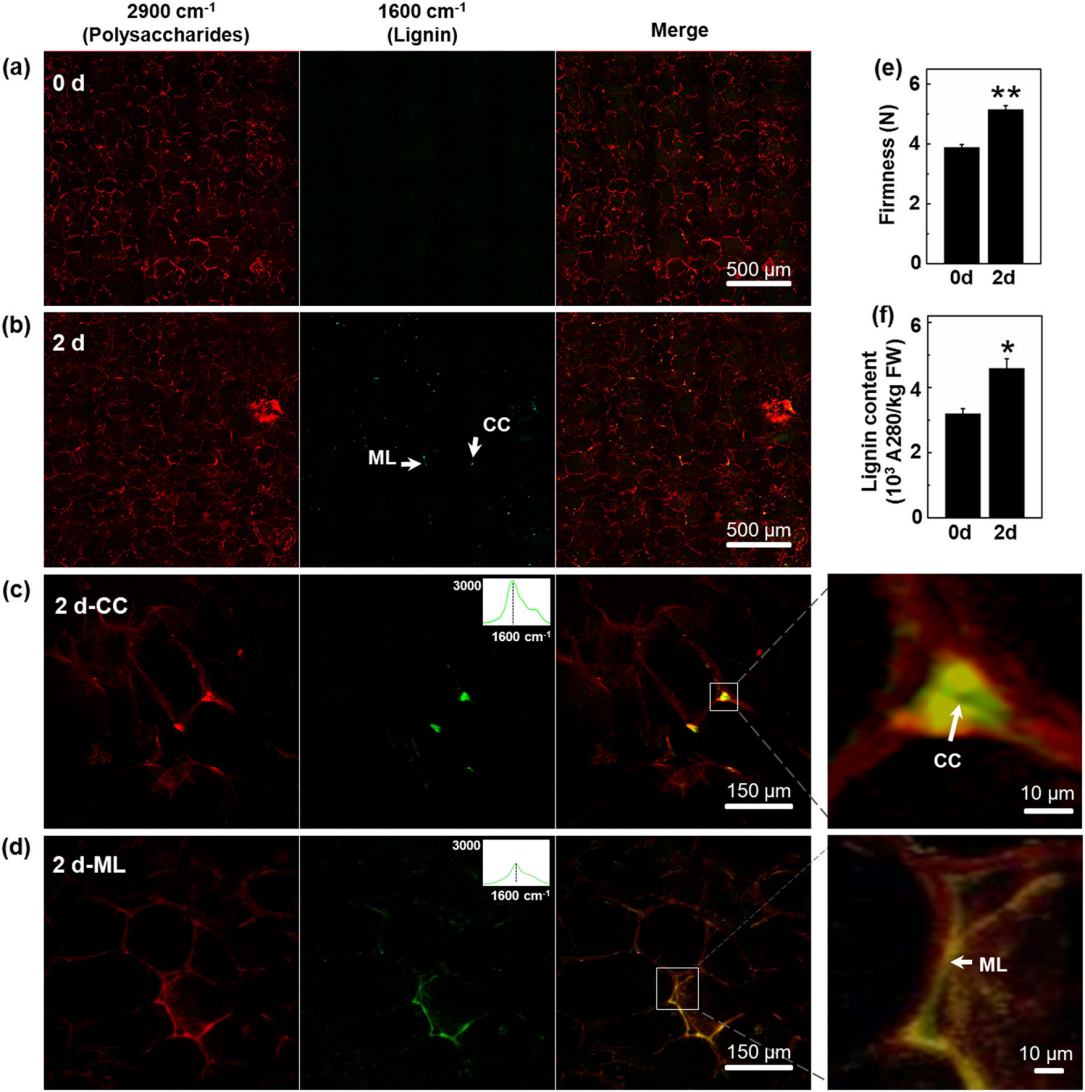
SRS imaging of the changes in the cell wall polysaccharides and lignin in the loquat flesh during storage at 0 °C. **a**, **b** Macroscopic SRS imaging of cell wall polysaccharides and lignin of loquat fruits at harvest (0 d) and after storage at 0 °C for 2 days (2 d). The lignins are deposited as spots in loquat flesh after 2 days of storage at 0 °C. **c**, **d** Microscopic SRS imaging of loquat flesh cells. The cell corners (**c**) and cell middle lamella (**d**) show lignin deposits. The upper right corners of (**c**) and (**d**) show the extracted spectra from the image stack. Enlarged views of CC and ML are shown to the right of (**c**) and (**d**), respectively. **e**, **f** The fruit firmness and lignin content of the loquats during storage

Previous studies have shown that the fruit firmness and lignin content of “Luoyangqing” loquat would increase obviously after 2 days of storage at 0 °C^11,25^. Therefore, we used loquat fruits collected at harvest (0 d) and after 2 days of storage at 0 °C as study materials to visualize the lignin deposition. Figure 2 shows SRS images of the cell wall polysaccharide and lignin distributions in loquat flesh during storage. From the macroscopic SRS imaging, the signals of cell wall polysaccharides at 2900 cm^−1^ are reduced after 2 days in storage (Fig. 2a), whereas the lignin signal at 1600 cm^−1^ is increased (Fig. 2b). The lignin deposits were observed as spots that were distributed in the loquat flesh. Notably, microscopic SRS imaging indicates that the lignin deposits are trapped in the cell corner (CC) (Fig. 2c) and middle lamella (ML) (Fig. 2d) of the loquat flesh cells during storage. The extracted spectra from the signal at 1600 cm^−1^ of the CC and ML are typical of lignin, showing a shoulder at 1660 cm^−1^. The SRS chemical imaging of loquat flesh indicates that lignin might be deposited in the CC and ML during postharvest storage at 0 °C. In addition, the physiological analysis indicated an increase in fruit firmness and lignin content after 2 days of storage at 0 °C. The fruit firmness was 3.88 N at harvest, and it increased to 5.14 N following 2 days of storage at 0 °C (Fig. 2e). The lignin content increased from 3.20 to 4.59 A_280_ kg^−1^ FW^−1^ (Fig. 2f).

### Topochemistry analysis reveals the compositional and structural differences in the LC, CC, and ML by spontaneous Raman microscopy

**Fig. 3:**
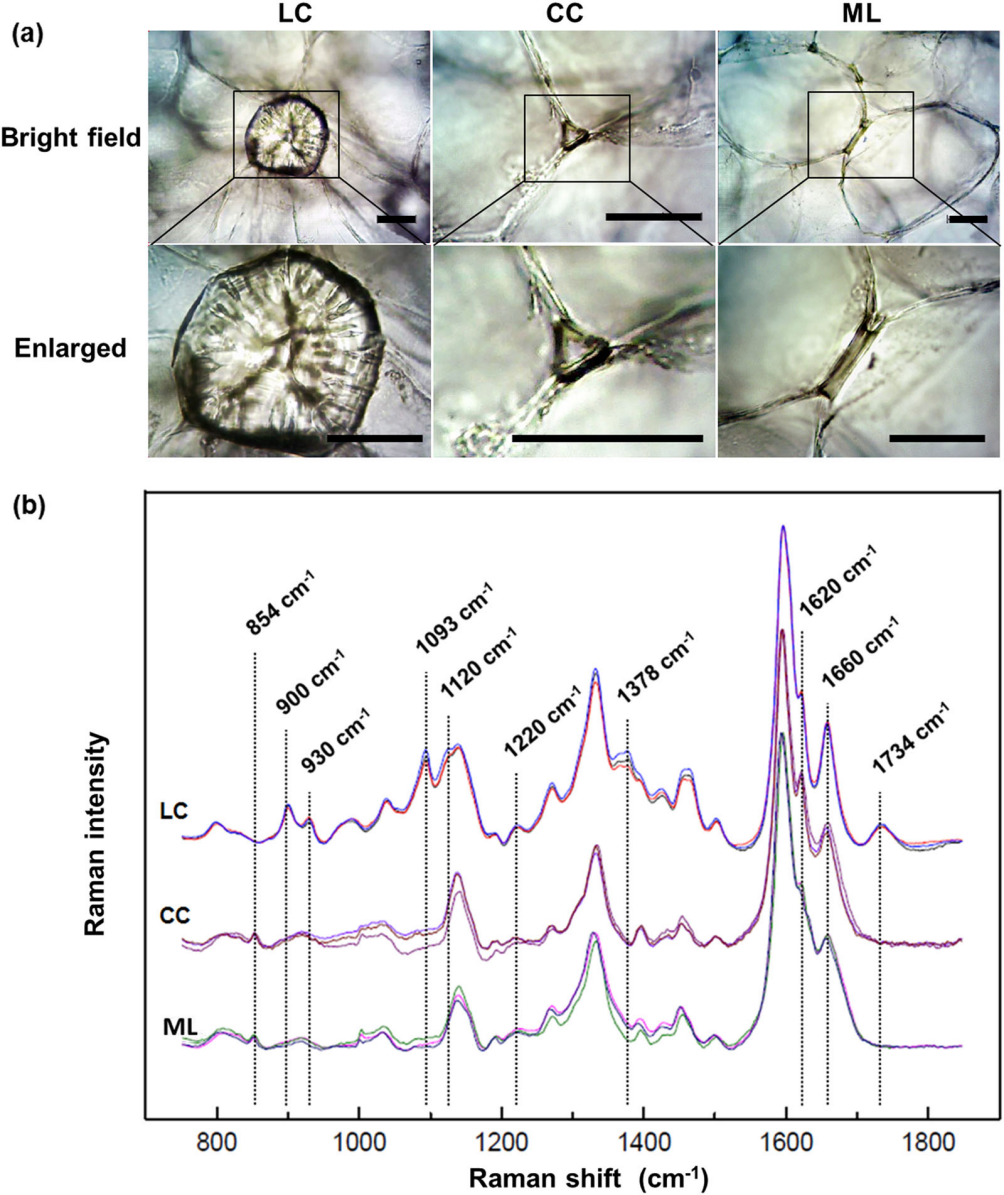
Comparison of the representative Raman spectra covering the entire window of molecular vibrations for the LC, CC, and ML in loquat fruits. **a** Bright field and enlarged images of the LC, CC, and ML. **b** The averaged Raman spectra of the LC, CC, and ML. The LC, CC, and ML each has three repeat data. Spectral differences among the LC, CC, and ML were primarily centered at 854, 900, 930, 1093, 1120, 1220, 1378, 1620, and 1734 cm^−1^. Scale bar: 50 μm

Figure 3a shows the bright field and enlarged images of the LC, CC, and ML in the loquat flesh. As shown, the LC has highly thickened walls filling almost all the cell volume. In the PC, the walls in the tricellular junction zones, i.e., the CC, are thickened. In addition, some ML regions of the PC show thickened walls. Figure 3b presents the full average Raman spectra of the LC, CC, and ML, covering the entire window of molecular vibrations from 700 to 1800 cm^−1^. By comparison, the Raman spectra of the LC, CC, and ML show clear differences, primarily in the bands centered at 854, 900, 930, 1093, 1120, 1220, 1378, and 1734 cm^−1^.

**Fig. 4:**
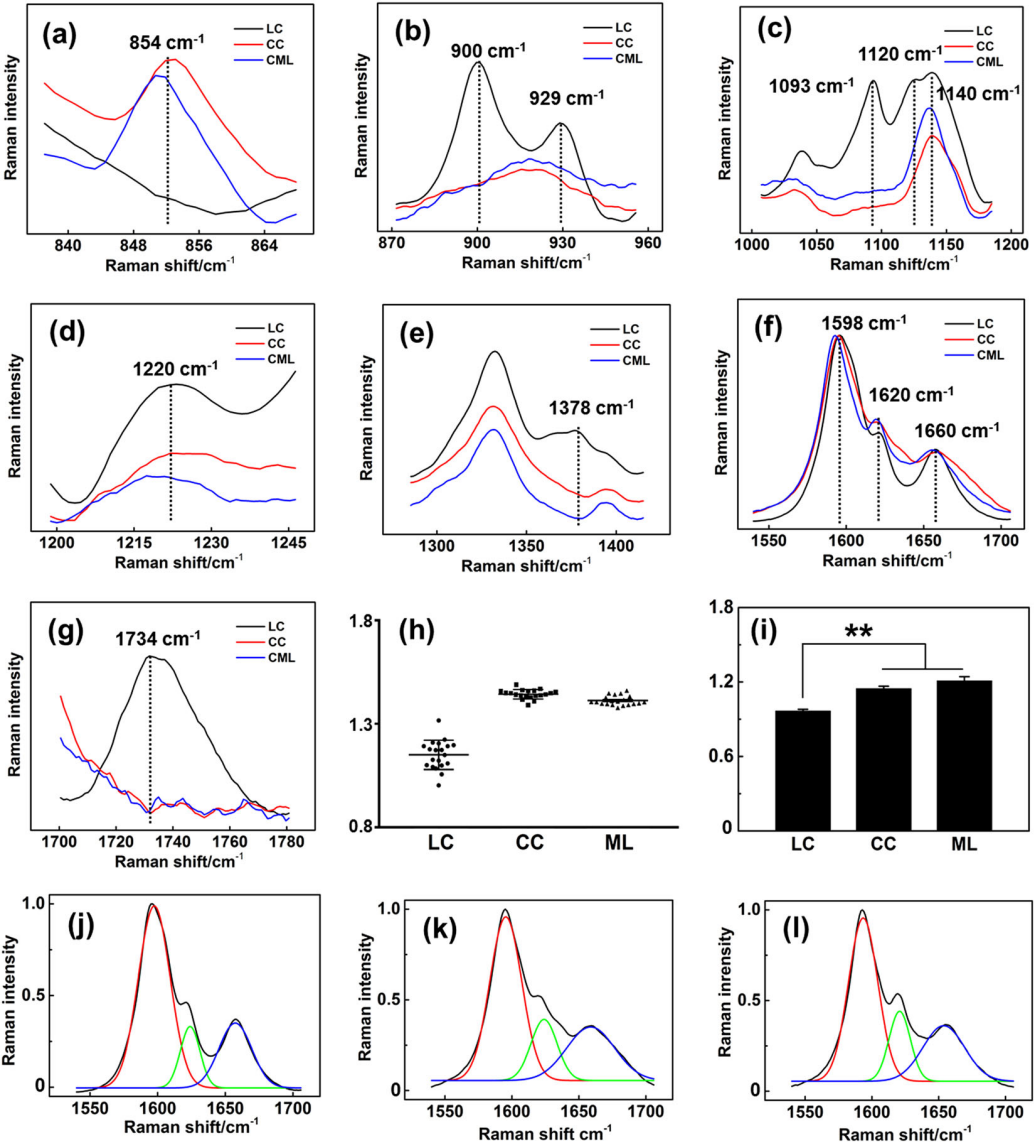
Detailed spontaneous Raman spectral comparison reveals the compositional and structural differences in the LC, CC, and ML in loquat fruit. **a** Spontaneous Raman spectral comparisons from 838 to 866 cm^−1^, **b** 875 to 955 cm^−1^, **c** 1010 to 1090 cm^−1^, **d** 1198 to 1247 cm^−1^, **e** 1270 to 1430 cm^−1^, **f** 1540 to 1710 cm^−1^, and **g** 1700 to 1780 cm^−1^. **h** The ratios of 1620 and 1660 cm^−1^ for 15 Raman spectra in each region (LC, CC, and ML) are randomly chosen for the ratio calculation. The error bars indicate the SE from 15 replicates. **i** The ratios of the Gaussian fitting peaks for 1620 and 1660 cm^−1^. The LC, CC, and ML each had three duplicates. The aldehyde and alcohol group ratios of lignin are higher in the CC and ML than in the LC. The error bars indicate the SE of three replicates. The asterisks indicate significant differences between the LC, CC, and ML: **P < 0.01. **j**–**l** Resolved Raman peaks of the typical Raman spectral region of lignin from 1540 to 1710 cm^−1^ for the LC, CC, and ML by multipeak Gaussian fitting

To explore the compositional and structural differences in the LC, CC, and ML in loquat flesh, the primary different regions in the Raman spectra were truncated and enlarged. As shown in Fig. 4a, the marker Raman peak of pectin centered at 854 cm^−1^ was detected in the CC and ML spectra, whereas this peak was not found in the LC spectrum. This result indicates that the CC and ML contain pectin, whereas the LC contains little. In the LC spectra, the peaks centered at 900 and 930 cm^−1^ are prominent (Fig. 4b). Both cellulose and hemicellulose contribute to the peak at approximately 900 cm^−1^ ^44^. The two peaks centered at 1093 and 1120 cm^−1^ are commonly used to investigate the nature of cellulose^34,45^. In the Raman spectra of the LC, both of these peaks were detected, but they were not found in the CC and ML spectra (Fig. 4c). Figure 4d shows the spectral comparison of the LC, CC, and ML from 1200 to 1235 cm^−1^, and the peak at 1220 cm^−1^ in this region is much more intense in the LC spectra. A recent study concluded that the 1220 cm^−1^ Raman peak is closely related to xylan^46^. In the spectral region from 1280 to 1430 cm^−1^, a peak centered at 1378 cm^−1^ is found only in the LC spectra (Fig. 4e). The 1378 cm^−1^ peak is attributed to cellulose and hemicellulose^44^. Figure 4f highlights the most typical Raman band region of lignin from 1550 to 1700 cm^−1^. The most prominent band centered at 1598 cm^−1^ is assigned to aromatic C=C vibration. Compared with that in the LC spectrum, the shoulder band at 1620 cm^−1^ is more intense in the CC and ML spectra. This shoulder band is assigned primarily to the ring-conjugated C=C stretch of coniferaldehyde/sinapaldehyde^33,37^. In addition, a broad peak at 1734 cm^−1^ is detected in the LC spectrum, but it is not found in the CC and ML spectra. This band was attributed to the C=O stretching of hemicellulose^37^. This result could provide evidence that the LC contains hemicellulose, whereas the CC and ML contain little. Figure 4h shows the intensity ratios of aldehyde groups to alcohol groups (1620 cm^−1^/1658 cm^−1^) of lignin deposited in the LC, CC, and ML. The intensity ratios of aldehyde groups to alcohol groups of the CC and ML lignin are much higher than that of LC lignin. In addition, the ratios are calculated based on the fitted peaks of the average Raman spectra by multipeak Gaussian fitting (Fig. 4i–l). The aldehyde group/alcohol group intensity ratios are also higher in CC and ML lignin (Fig. 4i). The relative intensity in combination with the absolute intensity (Fig. 4f) of aldehyde groups in lignin indicates higher incorporation of aldehydes into the CC and ML.

### Confocal Raman chemical imaging and topochemical compositional analysis of cold stored loquats

**Fig. 5:**
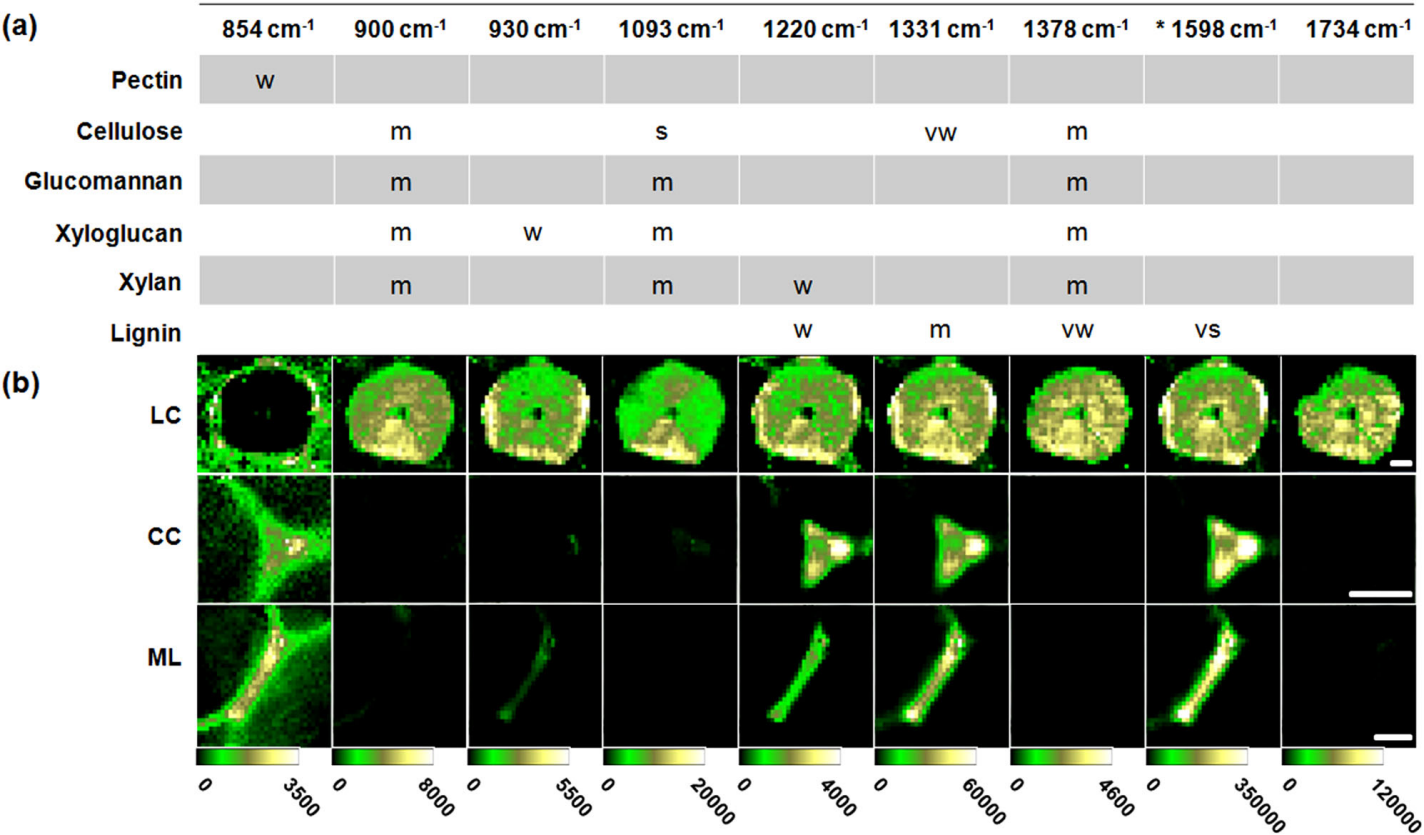
Attributions of the primary Raman peaks and the chemical imaging analysis of the LC, CC, and ML. **a** Attributions of the main peaks in the Raman spectra of the LC, CC, and ML. vw is very weak; w is weak; m is medium; s is strong; and vs is very strong. **b** Raman chemical images generated based on the hyperspectral data by integrating over the marker band regions of different components and functional groups. Scale bar: 20 μm

In contrast to SRS chemical imaging, spontaneous Raman images are generated based on the intensity or integral of specific peaks from the hyperspectral Raman data. In this study, the topochemical compositional characteristics of the LC, CC, and ML in loquats stored for 2 days at 0 °C were visualized by integrating over the primary band regions of different components and functional groups. Figure 5a shows the attributions of the primary peaks in the Raman spectra of the LC, CC, and ML from loquat flesh. Some Raman peaks are chemically specific because of the unique chemical groups in the measured components. In addition, some bands overlap due to the complex and multicomponent nature of plant materials. Considering the specificity of the peak at 854 cm^−1^ to pectin, this band is used to plot the distribution of pectin in the LC, CC, and ML of loquat flesh. As shown in Fig. 5b, the LC itself does not contain pectin, while the CC and the intercellular regions of LC contain pectin. The CC and ML of the parenchymal cells in loquat flesh are rich in pectin. In addition, some dispersive pectin was found to be distributed in the parenchymal cell lumen region that is close to the CC and ML. These pectin dispersions might be soluble pectin that was transformed from the insoluble pectin trapped in the CC and ML. Raman images generated based on the overlapping peaks of cellulose and hemicellulose, such as 900, 1093, and 1378 cm^−1^, showed differences among the LC, CC, and ML. The LC contained cellulose/hemicellulose, whereas the CC and ML contained little of either compound. According to the specificity of 930 cm^−1^ to xyloglucan, this band was used to plot the distributions of xyloglucan in loquats. The entire LC was found to be filled with xyloglucan, and the periphery showed a higher xyloglucan content than the inner part. The 1598 cm^−1^ peak is the Raman band most characteristic of lignin in plant materials. Using this band, the distribution of lignin can be mapped. The LC, CC, and ML contain lignin. The LC has a higher lignin content in the peripheral and central regions than in the intermediate region. The CC showed a higher lignin content in the conjunction regions. Raman images based on the other lignin peak centered at 1331 cm^−1^ showed a similar distribution to that of the 1658 cm^−1^ band. Considering that the 1220 cm^−1^ peak is closely related to xylan, this band was used to plot the distributions of xylan in LC, CC, and ML.

Xylan showed similar distributions with lignin in the LC, CC, and ML. It should be noted that lignin also contributed to this band. The distribution map of hemicellulose was constructed by hand at 1734 cm^−1^. The hemicellulose is distributed evenly in the LC.

Based on the abundant spontaneous hyperspectral Raman data, the topochemical compositional and structural imaging of the LC, CC, and ML in loquats was achieved. The LC was found to be a combination of lignin, cellulose, and hemicellulose. Compared with the PC, the LC has high lignin, cellulose, and hemicellulose contents. The formation of the LC might be a slow process through the thickening of the secondary wall of PC. In contrast, the CC and ML contain only lignin, without cross-linked cellulose and hemicellulose. The lignins in the CC and ML regions are more likely to be newly synthesized during cold storage.

### Raman imaging of functional groups in loquat lignin

**Fig. 6:**
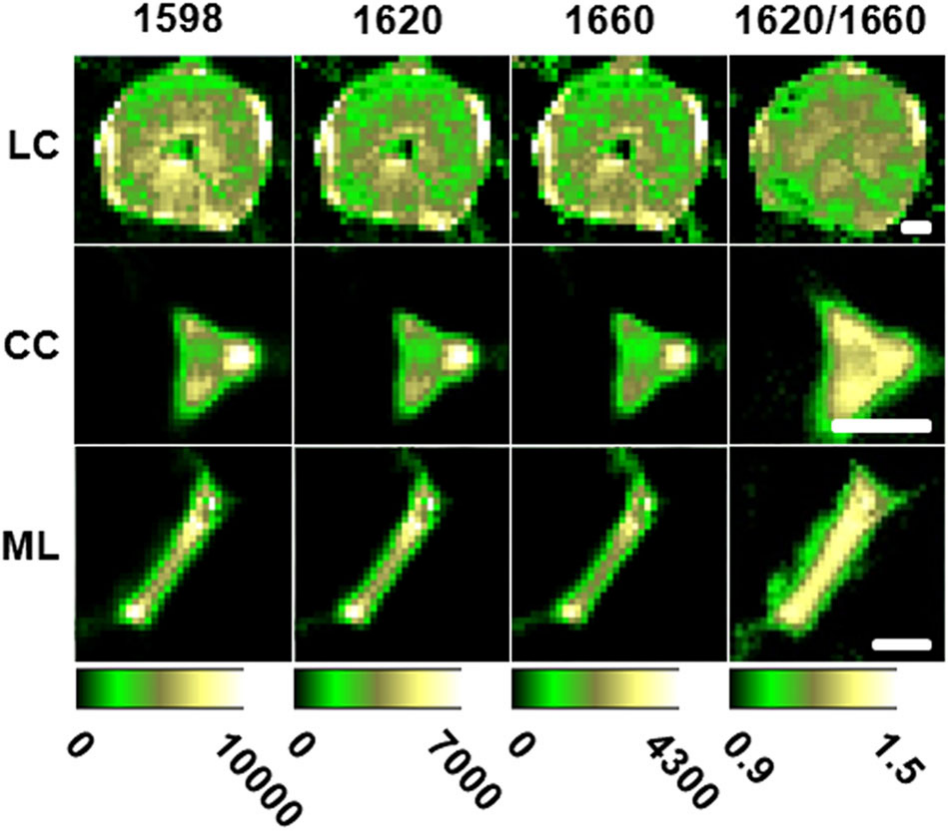
Raman images of distributions of different lignin functional groups in the LC, CC, and ML regions of loquats. Raman bands centered at 1598, 1620, and 1660 cm^−1^ are used to plot the distributions of the aromatic rings and aldehyde and alcohol groups, respectively. Images of the intensity ratio distributions (aldehyde groups: alcohol groups) in LC, CC, and ML are also plotted. Scale bar: 20 μm

The Raman signals are derived from molecular vibrations that reflect structural information, including the types of chemical bonds or functional groups. In the present study, the aldehyde groups and alcohol groups of lignin in loquats are visualized using the Raman intensities of the two shoulder peaks centered at 1620 and 1660 cm^−1^. As shown in Fig. 6, the images of aldehyde groups and alcohol group distributions show very similar patterns to that of aromatic ring vibration in each region. Nevertheless, the intensity ratios between the aldehyde and alcohol groups show differences among the LC, CC, and ML. Compared with the LC, the CC and ML exhibited higher aldehyde/alcohol ratios, which are visualized in Fig. 6. This result indicates that the CC and ML contain more aldehyde structures than the LC in the loquat flesh.

## Discussion

Softening and lignification are the two primary types of texture variations observed during fruit ripening and postharvest storage^1^. To date, fruit softening has been extensively investigated^3,47,48^. However, studies on flesh lignification, which hardens fruits, are relatively scarce. In this study, we investigated the deposition of lignin in loquats during lignification by using complementary SRS and spontaneous Raman microscopy. The findings in this study supplemented the spatial information on the lignin increase in loquat during postharvest storage.

In this investigation, macroscale chemical imaging using SRS was an attractive way to present the overall and comprehensive chemical distribution dynamics of loquat flesh during storage; thus, the overgeneralization caused by the narrow field of microvision was avoided. Here, the morphological and compositional heterogeneities of loquat flesh cells were observed within one comprehensive view by SRS chemical imaging (Fig. 1). The LC and VB were found to contain high lignin and cell wall polysaccharide contents. The PC showed cell wall polysaccharide SRS signals, but the lignin signal in the PC was scarce. In previous studies, the Raman peaks of PC cell wall polysaccharides in fruits and vegetables were primarily assigned to cellulose, hemicellulose, and pectins^8,49,50^, and very few studies have investigated the Raman characteristics of lignin in the PC walls. The reason might be the very limited or even absent lignin content in the PC walls in fruits and vegetables.

In plants, lignin biosynthesis begins in the cytosol with the synthesis of glycosylated monolignols from the amino acid phenylalanine^51^. The monolignols are then released into the initiation sites, i.e., the CC and ML, where the polymerization process begins^19^. In loquat fruits, the CC and ML were found to deposit lignin during postharvest lignification. Moreover, the CC and ML regions showed a higher incorporation of aldehydes into lignin. Biotic and abiotic stresses are proposed to promote the incorporation of aldehyde groups into lignin^52^. Therefore, the higher incorporation of aldehyde groups into lignin in the CC and ML of loquats might be a response to cold stress during storage at 0 °C. In this study, the SRS signals at 2900 cm^−1^ were found to be reduced. This observation might be attributed to the degradation of other cell wall polysaccharides, such as cellulose, hemicellulose, and pectins.

In this study, we wanted to visualize the deposition of lignins in loquats during cold storage using visualization tools from the tissue level to the cell level, which can promote a better understanding of lignification in loquats. Therefore, we used a chilling treatment so that the loquat fruit could suffer chilling injury, resulting in substantial flesh lignification, which is consistent with the real situation in the loquat fruit industry, in which cold storage can cause lignification.

Researchers have endeavored to investigate the mechanism of flesh lignification primarily in relation to the physicochemical and molecular biological characteristics. In particular, in terms of the transcriptional regulation of lignin biosynthesis during flesh lignification, transcription factors such as *EjMYB1*, *EjMYB2*, *EjNAC3*, and *EjNAC3*, together with their targets, have been identified^11,14,25,27^. These findings have greatly improved our understanding of lignification in loquats. Nevertheless, very few studies have addressed the cell or cell wall biology of flesh lignification. Our previous study revealed that a special type of cells, namely, lignified cells, which have high lignin content, were present in “Luoyangqing” loquats^32^. The cell-level visualization of lignin deposition has great significance for revealing its heterogeneity in loquat flesh. This visible heterogeneity in combination with single cell-omics analysis can further support a thorough investigation of the flesh lignification in loquats during postharvest storage. It should be noted that although chilling induces flesh lignification, which increases lignin content and firmness, which is in turn undesirable for consumers, it might be positive in helping the fruit survive low-temperature environments.

In conclusion, the deposition of lignin in cold-stored loquats was investigated from the tissue level to the single-cell level by using complementary SRS and spontaneous Raman microspectroscopy. Morphological and compositional heterogeneities among the massive cells in loquat flesh were found by SRS chemical imaging. After 2 days of storage at 0 °C, the CC and ML in the loquat flesh showed lignin deposits. The further detailed compositional and structural analyses of LC, CC, and ML by Raman microspectroscopy revealed that the LC was an integration composed of lignin, cellulose, and hemicellulose with pectin distributed around it, whereas the CC and ML contained only lignin and pectin without cross-linked cellulose and hemicellulose. Moreover, the CC and ML regions showed a higher incorporation of aldehydes into lignin. The above compositional and structural analysis suggests that the lignin in the CC and ML regions of loquats is later synthesized alone during storage. The findings revealed by complementary SRS and spontaneous Raman microspectroscopy provided a novel perspective on understanding loquat lignification.

## Materials and methods

### Plant materials and treatment

Fruits of the “Luoyangqing” loquat were harvested in 2018 from an orchard located in Luqiao, Zhejiang, China. The fruits were then transported to the laboratory on the day of harvest. Fruits of uniform commercial maturity, size, and color with no mechanical wounding or disease were selected and divided into two batches. One batch was used for experiments immediately, and the other was stored at 0 °C for 2 days and then used for subsequent experiments.

Each batch of the fruit consisted of 30 fruits. Ten fruits were used for measuring the fruit firmness, five fruits were used to obtain the cryosections of flesh for Raman imaging, and 15 fruits (three replicates, each contained five fruit) were cut into small pieces after the skins and stones were removed, then frozen in liquid nitrogen and stored at −80 °C for further use. Independent samples were used for the measurements of fruit firmness, firmness determination, and Raman imaging.

### Fruit firmness

The fruit firmness was measured using a TA-XT plus Texture Analyzer (StableMicro Systems, Surrey, UK) with a 5 mm diameter plane-head probe^16^. The penetration rate was 1 mm s^−1^ with a penetration depth of 4 mm. The maximum force value in the force-penetration curve was used to indicate the firmness. The firmness of each fruit was averaged from two measurements taken 90° apart at the fruit equator after the removal of a small piece of peel. The fruits were fixed by hand during the measurement to prevent rolling. Ten fruits were sampled at each sampling point. The fruit firmness was expressed in newtons (N).

### Lignin content determination

The lignin content was determined by the method of precipitation of lignin thioglycolic acid according to Xu et al.^25^. The frozen tissue was ground into a powder and 3 g was homogenized in 5 ml of washing buffer (100 mM K_2_HPO_4_/KH_2_PO_4_, 0.5% Triton X-100, 0.5% PVP, pH 7.8). The mixture was then stirred (250 rpm, 30 min) and centrifuged (5000*g*, 20 min) at 25 °C. The pellet was resuspended and washed twice in washing buffer as indicated above. A 100% methanol solution was used to wash the pellet four times, and it was dried at 80 °C for 12 h in an oven. Then, 10 mg of the dried residue was dissolved in 1 ml of 2 M HCl and 0.1 ml of thioglycolic acid and boiled in a water bath for 8 h. This reaction mixture was then cooled on ice and centrifuged (10,000*g*, 20 min). The pellet was washed twice with distilled water and dried at 65 °C for 12 h. The dried residue was dissolved in 2 ml of 1 M NaOH, with shaking (150 rpm, 18 h) at 25 °C and centrifugation (10,000*g*, 20 min) at 20 °C. A 0.5 ml volume of the supernatant was mixed with 0.1 ml of concentrated HCl. The mixture was left to stand for 4 h at 4 °C and centrifuged (10,000*g*, 20 min) at 4 °C. The precipitated lignin thioglycolic acid was then dissolved in 1 ml of 1 M NaOH. The absorbance at 280 nm was measured using 1 M NaOH as the blank. All measurements were performed in triplicate.

### Cryosection

Cryosections of loquat flesh for Raman chemical imaging were obtained according to Zhu et al.^32^. Flesh from the equatorial region of the fruits was cut into cubes (5 mm × 5 mm × 5 mm) and embedded immediately using optimum cutting temperature (OCT) freezing medium. After the embedded flesh cubes were frozen, they were mounted onto the “specimen disc” and sectioned into thick sections at a thickness of approximately 100 μm using a freezing microtome (Leica, Wetzlar, Germany). The temperature inside the chamber of the microtome was kept at −15 °C during sectioning. The sectioned tissues were washed with distilled water to remove the OCT freezing medium. The sections were then placed on a glass slide with a drop of water and covered with a 0.17-mm-thick coverslip.

### SRS microscopy

SRS chemical images were constructed based on a parallel two-color SRS microscope with the two orthogonal outputs of a dual-phase lock-in amplifier (LIA) as described previously (dual-phase SRS). The pulsed femtosecond laser beams from a commercial optical parametric oscillator (OPO) (Insight DS+, Newport, CA) were used as the excitation laser sources. A fundamental 1040 nm laser with a pulse duration of 150 fs was used as the Stokes beam, and the tunable OPO output (690–1300 nm, 120 fs) served as the pump beam. The two laser beams were combined using a dichroic mirror (DM), spatially and temporally overlapped, and then allowed to interact with the samples through a laser-scanning microscope (FV 1200, Olympus). To capture the Raman frequencies at 2900 cm^−1^ for cell wall polysaccharides and 1600 cm^−1^ for lignin in loquat flesh, the tunable outputs were turned to 802 and 892 nm, respectively. A 60× water immersion objective lens (NA = 1.2, UPlanApo/IR, Olympus) was used to focus the light into the sample, and an oil condenser (NA = 1.4, U-AAC, Olympus) was used to collect the signal. Both the reconstruction of large-scale images and the extraction of SRS spectra from the image stacks were performed with ImageJ software (National Institute of Health, Bethesda, MD, USA).

### Spontaneous Raman spectroscopy

The spontaneous Raman hyperspectral data were collected with a Renishaw inVia Reflex confocal Raman Microscopy System (Renishaw Plc., Wotton-Under-Edge, UK). This system is equipped with a 532 nm diode laser, an air-cooled charge-coupled device (CCD) and a Leica DMLM microscope. The hyperspectral imaging data were acquired under the 50× objective with a step size of 2 μm for both the *x* and *y* directions. Each collected spectrum ranged from 600 to 1800 cm^−1^ in static mode, with an exposure time of 1 s, a laser power of 25 mW and 3 time accumulations. The collected raw hyperspectral data were processed using Renishaw Wire 4.3 software. First, the nearest neighbor cosmic removal method was used to remove the cosmic rays. Then, by using the noise-filtering function, the level of random noise in the dataset was reduced. In the noise filtering function, principal component analysis was applied to the dataset to load the valuable spectral information. Third, the baseline from the raw dataset was corrected by using the intelligent fitting mode of the subtract baseline function with a polynomial order of 8 and a noise tolerance of 1.20.

The representative average Raman spectra of the LC, CC, and ML were obtained as follows. Using LC as an example, the Raman spectra of 15 pixels inside the LC region were randomly, evenly, and manually selected using Renishaw Wire 4.3 software to calculate the averaged Raman spectrum of the LC. In addition, the Raman spectra of 15 pixels in the background region (cell lumen) were also randomly and evenly selected and used for calculating the average Raman spectrum of the background (primarily glass signals). The representative Raman spectrum of the LC was then obtained by using the averaged Raman spectrum of LC to subtract the averaged background spectrum. The calculation of representative LC Raman spectra was performed for three biological replicates. The representative Raman spectra of the CC and ML were obtained in the same way as the LC. The averaged Raman spectra of the LC, CC, and ML in Fig. 4 were obtained by averaging the three biological duplications of their representative Raman spectra.
